# The Potential of a Thick Present through Undefined Causality and Non-Locality

**DOI:** 10.3390/e24030410

**Published:** 2022-03-15

**Authors:** Alessandro Capurso

**Affiliations:** Independent Researcher, 00185 Rome, Italy; ale.capurso@gmail.com

**Keywords:** time, presentism, causality, free will, information, logic, entanglement, non-locality, CTC

## Abstract

This paper elaborates on the interpretation of time and entanglement, offering insights into the possible ontological nature of information in the emergence of spacetime, towards a quantum description of gravity. We first investigate different perspectives on time and identify in the idea of a “thick present” the only element of reality needed to describe evolution, differences, and relations. The thick present is connected to a spacetime information “sampling rate”, and it is intended as a time symmetric potential bounded between a causal past of irreversible events and a still open future. From this potential, spacetime emerges in each instant as a space-like foliation (in a description based on imaginary paths). In the second part, we analyze undefined causal orders to understand how their potential could persist along the thick present instants. Thanks to a C-NOT logic and the concept of an imaginary time, we derive a description of entanglement as the potential of a logically consistent open choice among imaginary paths. We then conceptually map the imaginary paths identified in the entanglement of the undefined orders to Closed Time-like Curves (CTC) in the thick present. Considering a universe described through information, CTC are interpreted as “memory loops”, elementary structures encoding the information potential related to the entanglement in both time and space, manifested as undefined causality and non-locality in the emerging foliation. We conclude by suggesting a possible extension of the introduced concepts in a holographic perspective.

## 1. Introduction

The nature of Time is often at the root of the debate in physics and possibly sits at the core of the General Relativity (GR) and Quantum Mechanics (QM) incompatibility. 

In recent years, the search for a theory of Quantum Gravity (QG), able to include the success of both GR and QM, revived the study of time as a key ingredient for the understanding of a quantum description of spacetime.

After an investigation on multiple perspectives on the subject, this paper suggests the interpretation of time through the concept of a time symmetric “thick present”. Within each thick present instant, intended as the only element of reality along an emerging axis of a thermodynamic and causal time, a quantum information potential ***T_k_*** is considered, from which spacetime emerges in a sequence of space-like foliations.

Beside time, the concept of entanglement has puzzled the physics community for decades, stimulating the discussion around causality and locality. 

In an evolution occurring in discrete instants, we investigate how indefinite causal orders (as entanglement in time) could be intended.

We first consider undefined causality through a parallel with a C-NOT quantum gateway. Following a path integral approach, we then describe the information in the undefined order through entangled imaginary paths in the C-NOT circuit, which develop as superposed imaginary times in each space-like foliation. 

The superposition of imaginary times in a time-symmetric potential is finally interpreted as a closed path (CTC) in the thick present.

In the context of a Quantum Information Science (QIS) description of spacetime, CTC@*T_k_* are interpreted as logically consistent “memory-loops”, in which the information potential of an “open choice” (temporal order or spatial position) persists along the succession of the present instants. 

We conclude by conjecturing a connection between entanglement (*EPR*), non-locality (*ER*), and CTC in the thick present through a holographic perspective.

## 2. Existence in a Thick Present

### 2.1. Understandings on the Nature of Time

The nature of time has often led to confusion, as several different meanings have been associated to the word “time”, both from neuroscience and physics, as illustrated by Buonomano and Rovelli in Ref. [1]. The debate is often reduced to two opposite and extreme interpretations. The first, called *Eternalism*, considers an ever-existing time, real in both the past and the future, and of which we perceive only limited *cones* of information. The opposite perspective, known as *Presentism*, pictures time as a vanishing present compressed between past and future instants. The focus is on the difference between the past, already happened and irreversible, and the future, still undefined and open.

Neuroscience considers a forward and causal progression of time: a fixed past and an undefined future, both not-existing, as only the subjective experience of “here and now” is given as “real”. Our neurons equalize different asynchronous stimuli and derive a coherent picture of the surrounding space in each “perceived instant”, remembering the past events. Our perception is not always physically accurate but to our brain looks coherent, and this is enough for a perception of a “flowing time”: a memory of the past connected to the future through what is happening in the present. It could be considered a *local presentism*, with no interest for the idea of time at quantum or cosmological scales, but the wish to reconcile the human perception with more fundamental phenomena.

Physics, from the perspective of thermodynamics, considers the arrow of time as “emerging on average” or macroscopically (but not fundamental). Newtonian mechanics is compatible with a *global presentism*, as a “perceived now” common to all entities, but the relativity of time in GR seems to preclude this possibility. According to GR, time is described as intertwined with space (in a relativistic spacetime), but space and time are different concepts anyway, related to a clearly identifiable “space separation” between events (no causal influence) or “time separation” (possible causal influence).

Even if GR is our best theory on spacetime, we should consider its insights with caution: as a classical theory, GR should at least be incomplete. QG approaches are investigating *time-less* scenarios, and further studies may show that a relativistic description of time is emergent from more fundamental principles.

Finally, Planck units strongly suggest a discreteness of space and time, highlighting the existence of absolute references derived from fundamental constants in our universe.

From a QM and a logical perspective, in the context of the *Free Will theorem* (introduced by Conway in Ref. [2]), we should assume that the future is undetermined and posse a serious threat to the idea of a classical and ever-existing time in a “block-universe”. As Freeman Dyson wrote in [3], the only role of a local observer seems to be the distinction between a classical past and a probabilistic future. The relation between the observation of a variable at a given instant (identifying an event) and the derived difference between past and future has also been elaborated by Smolin in Ref. [4] as a *dynamics of difference*.

From a QIS perspective, recent *gargantuan simulations* (reported in Ref. [5]) showed that time seems irreversible at the most fundamental level, beyond thermodynamic reasons. It has been shown that even a simple three-body system “*would require an accuracy of smaller than the Planck length in order to produce a time-reversible solution*”. 

Eventually, what has already been can causally influence the present but cannot be changed and, beyond fundamental physical limits, cannot even be known with certainty.

The irreversibility of events might seem to be in contradiction with a symmetric description of time, even if physical laws in a classical framework are time-symmetric and there are several QM symmetric approaches. The idea of an emerging reality connected to the superposition of both a forward and a backward propagating wave goes back to the fifties, introduced by Watanabe in Ref. [6] as Two-State-Vector Formalism (TSVF).

In recent years, in the context of a time-symmetric approach, the concept of irreversibility has been more clearly connected (in Refs. [7,8]) to the idea of *irretrodictability* from a logically consistent Bayesian perspective. The emergence of a causal arrow of time from a more fundamental requirement of logical consistency has also been investigated in [9]. Additional insights on a time-symmetric description with elements of Energetic Causal Sets has been developed by Cohen et al. in Refs. [10,11], further smoothing the tension between a causal and irreversible perspective irremediably opposed to time symmetry.

### 2.2. Identifying a Quantum of Evolution

If the future is open and yet to come, the past is irreversible (might not even be known beyond certain limits) and time shows a level of symmetry in its evolution, we should then consider a time-symmetric thick present as the elementary quantum in the passage of time.

We can define a thick present as the information ***T_k_*** related to a thick space-like foliation, bounded by −*T* and +*T* and derived from a time-symmetric superposition of perspectives (from a near past and a near future) on the emerging spacetime.

Within a thick present we can consider both the quantum information potential (in a time-symmetric description) as well as the information of the last events (intended as causal points beyond the past boundary of the thick present, from which the present emerges and the future opens), efficiently discarding (for Occam’s sake) all the information that is not “currently needed” to describe the evolution of the Universe. A thick present is the actual realization of a discreteness of time and, from an ontological perspective, shall be intended as the only element of reality in a logically consistent, causally and thermodynamically oriented emerging axis of an extended classical time.

It is worth to note that, as highlighted by Tallant and Ingram (Ref. [12]), a well-defined philosophical framework of *Presentism* is missing, as several (and sometimes contradicting) descriptions are proposed in the literature. Among them, we will consider in this contribution the definition stating that “*Only the present time exists (No non-present time exists)*”.

Following a philosophical perspective grounded on the physics of irreversible events, open future and indeterminate present, a *Presentism* interpretation of time has also been recently promoted by Mariani and Torrengo in Ref. [13].

In the search for a quantum description of space and time, a thick present has been considered by Gisin in Ref. [14] (via an intuitionist mathematical language), and by Smolin in Ref. [15] (from an ontological perspective in QM).

The concept of an everchanging *becoming* between a fixed past and a probabilistic future has also been investigated by Schlatter. Starting from a principle of synchronization, the gravitational potential is connected in [16] to a foliation of spacetime in space-like surfaces and, sequencing the *flow of reality* in time intervals, the established relations between energy, entropy, and geometry are recovered. In Refs. [17,18], events are interpreted through synched *light clocks* (introduced in Ref. [19]) in an emerging *thermal time*, and evolution is intended through a *realm of probability amplitudes* (with a symmetric time structure) and an emerging *empirical spacetime* (as events break the unitary symmetry).

A thick present can be interpreted in QIS as a discrete elaboration of the global information potential in the space-like foliation. 

There are several theories that consider evolution in discrete steps. To mention a few, *Finite State Classical Mechanics* (described by Margolus in Ref. [20]) is based on Lattice Dynamics, where evolution rules are often referred to as *cellular automata* models. *Signal-State Quantum Mechanics*, developed in a theory of *Quantized Detector Networks* (presented in [21]), is a realization of the Heisenberg’s “instrumentalist approach” to quantum physics.

Following the insights of QIS and “*it from bit*” (that considers spacetime and QM as emerging from a quantum information processing), Operational Probabilistic Theories (OPT), developed by Hardy, D’Ariano, et al. (Refs. [22,23,24]), describe the evolution of quantum systems as logical-physical circuits that can be foliated in hyper-surfaces elaborated in atomic steps. OPT have been considered in a time-symmetric perspective (Refs. [25,26]) and in terms of a difference between *known* and *unknown*, rather than an emerging past and future (as in Ref. [27]). Even if the idea of a thick present has not been considered yet in the context of an OPT description of spacetime, we can identify in the atomic processing of OPT the realization of an atomic thick present and then map the space foliation emerging in each cycle to an equivalent circuit-foliation.

To describe a time-symmetric thick present in the context of a discrete evolution of the information (phased on atomic cycles), we will consider a minimum time interval *T* like a *π* rotation, and 2*T* for a full cycle in a time-symmetric description. We will interpret these discrete 2*T* steps, from (2*k* − 1)*T* to (2*k* + 1)*T*, as the duration of the atomic elaboration of the present potential ***T_k_*** from which spacetime emerges as a space-like foliation at 2*kT*.

It is worth to clarify that the concept of “*spacetime from information*” is not promoting the idea that “*we live in a simulation*”, which is an unneeded speculation. Moreover, the “*present*” is not intended as a “global perceived now”. The passage of time for local systems follows relativity, and time intervals measured by quantum clocks (even through events) vary according to GR, as there is no absolute perspective for any local observer within the emerging spacetime, but only relative ones. The “perceived now” of quantum systems (from particles to complex clocks), shall be intended as a “proper evolution cycle” of the system, measured on to the past experienced cycles and of greater duration compared to the thick present extension, as the spatially non-local “here” that spreads in the wave-function.

The thick present ***T_k_*** represents the potential of the *k*th space of events and possibilities in a space-like foliation of our universe bounded within (2*k* − 1)*T* and (2*k* + 1)*T*. In a QIS picture, its duration 2*T* is intended as a spacetime information “sampling rate”. The idea of a maximum rate of change connected to the inverse of the Planck time has been elaborated in Ref. [28], where it has been proven to be compatible with Relativity.

We will consider the present cycle duration as a global reference for time intervals to allow a relative confrontation of quantum clocks with respect to one another in a discrete passage of time. Eventually, even a “*Relational time*” (defined in [29] as the “*counting of happenings*”) needs an “elementary event” to allow independent clocks to compare their “*counts*” in a coherent and consistent way, as absolute references are always required for uniformity in comparisons. In this sense, the present atomic processing cycle can represent a quantum of elementary action (the “fastest event” to evaluate differences) available at every point in spacetime as a reference on which any relational and relativistic perspective can rely.

Observers, events, or potential events all coexist in a thick present able to account for a superposition of perspectives on the information from a near past and a near future, being atomic and time-symmetric within its thickness, and assuring consistency between “what it was” (causally happened) and “what it could be” in the current cycle.

The information persists and evolves as a potential in case no specific events occurred in the present elaboration cycle, while events of decoherence shall be considered irreversible, in line with a QIS description and the causal set perspective. From the irreversibility of events, a thermodynamically oriented arrow of time in line with causality can also be considered as emerging in the succession of the thick present instants.

### 2.3. Conclusion on Presentism and Open Challenges

A *Presentism* perspective on time has ancient origins, it is rooted in several western and eastern philosophies, and it is coherent with the latest interpretations in neuroscience.

Relativity, allowing for a description of reality focused on local observers, has then taught us that every local description could and should be relative, dissolving an absolute passage of time in a relativistic spacetime fabric. The multiple relative perspectives on the same information in terms of events have mined the concepts of before and after, leaving to an absolute speed of propagation of the causal information the role to preclude paradoxes. Eventually, in a relativistic description, time appears in an extended *classical reality* of past events and a deterministic future, seemingly excluding a *Presentism* description from every interpretation in physics.

Recently, in the context of relativistic time intervals but beyond the limits of a classical description, part of the research community has tried to reconcile the interpretation of time with the ancient philosophical understandings, realizing that in the indeterminacy of the quantum information lives the potential of the present instant: it is the quintessence of any process, it causally depends on the set of past irreversible events and it is the door to an open future.

In the first part of this contribution, we have presented the main insights towards a description of time coherent with relativity and QM and with a *Presentism* perspective. In the context of QIS, we have proposed an interpretation of the present as the information in the *k*th evolution cycle of the space of events and possibilities, emerging as a space-like foliation, bounded between (2*k* − 1)*T* and (2*k* + 1)*T* in a time-symmetric description.

The temporal extension of the thick present, the actual realization of a discreteness of time, has been interpreted as a spacetime information “sampling rate”. The atomic elaboration of the information potential in each instant has been proposed as a quantum of elementary action, the absolute reference needed among independent observers or clocks to consistently define and compare any relativistic perspective on the *happenings* in the evolution of information.

In the framework of a thick present, Classical is then what we remember or causally predict, it is what has already happened or will happen if there were no quantum features. Complex observers can encode in the complexity of their internal dynamics the information of the past events and derive a corresponding thermodynamic and causal orientation of a classical time.

Classical reality emerges from the information encoded in the memory of complex observers but should not be intended as “currently real”: in an ontological sense, it does not exist. From the proposed perspective, the Universe exists in the thick present only.

**Figure 1:**
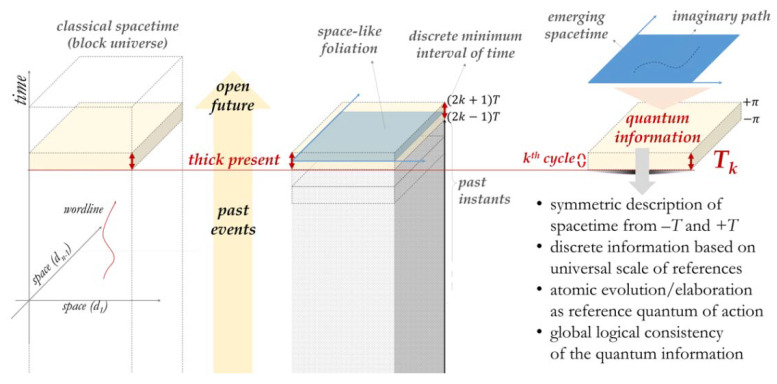
Identification of the thick present as the current thick space-like foliation and corresponding *k*th elaboration cycle of the quantum information ***T_k_***, from which spacetime is considered to be emerging.

Figure 1 graphically illustrates the introduced proposal.

In the given interpretation, the information ***T_k_*** plays a crucial role in the emergence of spacetime, and a better description of how this potential is encoded in the thickness of the present is needed. Moreover, recent experiments have shown that undefined causality (entanglement in time order) is possible.

In the following chapter we will investigate the relation between causality and logical consistency and propose an explanation as to how the information potential of entanglement in time orders could be intended and persist along the succession of the thick present instants. In the final part of this paper, we will conjecture a possible description of the information potential as undefined locality and entanglement in space from a holographic perspective.

## 3. Undefined Orders in Imaginary Times

### 3.1. Causality and Logical Consistency

Recent investigations in the physics of time highlighted the possible existence of Undefined Causal Orders (UCO) and derived the equivalent Bell’s inequalities in terms of temporal orders (as illustrated by Brukner et al. in [30]). The entanglement of temporal orders and the experimental verification of UCO have been considered as well in [31,32,33].

In an evolution described as occurring in thick present instants (given as the only element of reality in time), it is worth understanding how UCO could occur and how their potential, in the entanglement of temporal orders, could be intended.

The authors of Ref. [32] identify a “quantum SWITCH” circuit able to selectively choose the route of a particle, so that *Alice* (*A*) is encountered along the path before *Bob* (*B*) or vice-versa, depending on a controller qubit *C*.

The quantum SWITCH circuit can be described, from a logical perspective, as a device able to investigate the alternative scenarios “*A* happens before *B*” or “*B* happens before *A*”, equivalent to “*A*(*B*) is first in time and *B*(*A*) is not first”, through a controller qubit which is in a superposition of states.

In circuit logic, the same behavior can be described through a XOR function *A*⊕*B*, given that “*A*(*B*) is true” when “*A*(*B*) is met first on the path”. Given *A* and *B* as any pair of points along the path of a particle entering the circuit, the XOR gate superposes the two statements “*A* is first, and *B* is not first” and “*B* is first, and *A* is not first”. The resulting information of the XOR function is true if one and only one of the two assumptions is true, excluding the under-determined scenario (both false, as if there were no “first”) or, on the other hand, an over-determined solution (both true, as if both were “first”).

The XOR gate assures the logical consistency of the global information in the context of an “open choice”, limiting the possible outcomes to the only ones which imply a difference, and excluding the logical paradoxes of over/under-determined solutions.

In quantum logic, the XOR function can be described as a Controlled-NOT (C-NOT) quantum gate, which operates on a quantum register consisting of 2 qubits, *C* and *S* (Controller *C* and Target *S*), and flips the qubit *S* if and only if |C⟩=|1⟩.

The C-NOT gate correlates the information potential of the controller qubit *C* with the information potential of the Target *S* (the particle entering the circuit), and it is a common system used to create entangled pairs. For instance, an experiment in which a particle changes one of its quantum properties depending on the direction of travel in the circuit would create an entanglement between the observable in the particle and in the controller qubit, as if the potential information of the path of the particle were locally “stored” in *C.*

A similar description of the quantum SWITCH is given in [34] as a *quantum time flip* device. The authors elaborate the proposal in the context of OPT and unitary operations, implementing it through entangled photons.

**Figure 2:**
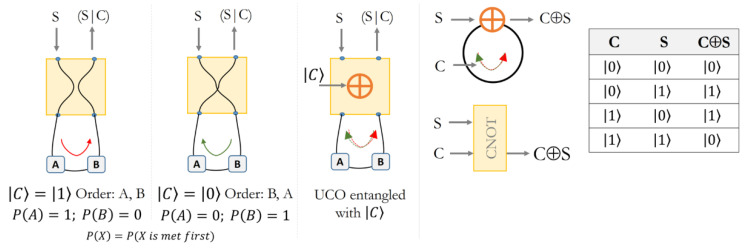
Controlled quantum SWITCH reproducing an UCO described as a XOR function, implemented as a C-NOT quantum gate. The entanglement of the Controller qubit *C* with the Target *S* at the point ⊕ allows the superposition of paths in which “*A*(*B*) is met first and *B*(*A*) is not”, and consequently the UCO. The entanglement in the C-NOT gate can be seen as the information potential of a choice instantiated in the instant of the interaction and of which the answer is undetermined.

It is crucial to highlight that, given the quantum superposition of the 2 circuit paths along the opposite directions, the “choice” could not happen immediately, at the time of the first *C-S* interaction. We need a measurement event to have a definite and irreversible outcome of the choice and, as long as the particle or the controller is not observed (and contextually defined), both results are possible: the outcome of the choice is a quantum information potential encoded in the relation between *C* and *S*, as illustrated in Figure 2.

### 3.2. Imaginary Closed Paths

To picture an undefined order within a thick present and the corresponding foliation, we can describe the path of the particle inside the circuit as a function of the propagation velocity v and of an imaginary time of motion needed to traverse the circuit (as a dimension of possible freedom), in a resulting imaginary path $iv\tau_{\left|C\right\rangle }$ along the circuit.

We will consider the point *C* as the position of the controller qubit closing the circuit in the space-like foliation. The point *A* is defined as the imaginary point reached at the imaginary time $i\tau_{\left|C\right\rangle }$ given a propagation along the circuit in the anticlockwise direction ($i\tau_{|C=1\rangle }$), while the point *B* is reached after the same time when moving in the clockwise direction ($i\tau_{|C=0\rangle }$). The point of entrance of the particle in the circuit (as the instant of entanglement with the controller *C*) becomes the point in the past of the particle and of the controller in which an information potential related to a logically consistent “open choice” was established.

The concept of an imaginary time has been popularized by Hawking in Ref. [35] and can be interpreted as a Wick rotation (able to offer a Euclidean description of the Minkowski metric) which is common in the Path Integral (PI) formulation of QM. In each instant, a space-like foliation can be described from the information in the causal set of events *F* (“fixed past choices” already defined at the past boundary of the present), in the points of quantum interactions generating potential *O* (as new “open choices”), and through an imaginary time of motion (as imaginary paths) emerging from them.

Considering an imaginary time of motion (*it_F,O_*) needed at the speed of light from any quanta of space, the imaginary paths traced along (*ict_F,O_*) (or *ict*, for short) define and trace an imaginary Minkowski space within the thick present.

A possible description of spacetime as space-like foliations in a PI context can rely on the definition of a new Hilbert space ℋ built upon the tensor product of copies of the conventional Hilbert space ℌ, one for each elaboration cycle ($ℋ∶={⨂}_{k}{ℌ}_{k}$), and then on the application of the related unitary time translation operator along the successive slices, as elaborated in Ref. [36]. The description of a relativistic particle in a PI formalism has been discussed in [37], where the Feynman propagator has been connected to the imaginary action in the space-like paths, to be accounted in the sum of all possible trajectories together with the *orthochronos* paths (at the speed of light).

Even if far from a proper derivation in a PI formalism, we could still consider the imaginary path $iv\tau_{\left|C\right\rangle }$ (in entanglement with the controller *C*) defined after the time of traversal of the C-NOT circuit as the representation of the quantum information connected to the open choice established in the undefined causality.

Modeling the entanglement in the undefined orders through a C-NOT quantum logic and superposed imaginary paths developing in an imaginary time of motion in opposite directions along the circuit allows a novel interpretation of the phenomena.

**Figure 3:**
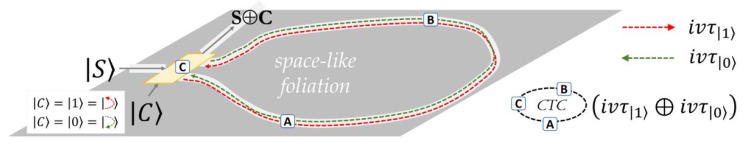
Superposition of the imaginary paths of the particle $\left(iv\tau_{\left|1\right\rangle }⊕iv\tau_{\left|0\right\rangle }\right)$ in a space-like foliation at a given instant after the time of traversal Δ*τ*. The result can be described as the superposition of a forward- and backward-evolving wave in the imaginary time of motion $\left(iv\tau_{\left|C\right\rangle }\right)$ and a CTC closed between *C* and any two points A and B on the circuit.

From the perspective of an imaginary time developing in the time-symmetric thick present, the superposition of the paths $\left(iv\tau_{\left|1\right\rangle }⊕iv\tau_{\left|0\right\rangle }\right)$ can be indented as the logically consistent superposition of a forward- and a backward-evolving wave persisting in the circuit, as well as an imaginary Closed Time-Like Curve (CTC) connecting the points *A*, *B,* and *C* (Figure 3).

The suggested relation between CTC in the thick present and entanglement is open to several interpretations and needs further clarifications, left to the coming chapters.

## 4. CTC, Entanglement, and Non-Locality

### 4.1. The Potential Hidden in a Choice

CTC have been studied since the early days of GR as possible solutions to Einstein’s field equations (Gödel in [38]), and their existence often raised concern (as in [39]).

Following the interest for a quantum description of GR, the nature of time gained momentum in the physics’ research of the recent years, together with the CTC puzzles.

It has been shown that CTCs are incompatible with a causal and thermodynamic progression of time and that events cannot happen along their path (further elaborated in Refs. [40,41,42]). Nevertheless, if time (as a progression related to causal events) cannot be considered on a CTC, the idea of a CTC developing along superposed imaginary times (within the space-like foliation) can represent the information potential of the system in the thick present. In the proposed interpretation, the superposed possibilities in the entanglement are considered as the information of a logically consistent “open choice”, discarding under/over-determined solutions in the XOR that encodes to the choice. This information is encoded in the CTC@*T_k_* as a potential of superposed values of the outcome of the choice and eventually a state with no identification of any event. In this sense, a CTC developing in the imaginary time represents an undefined causality in the thick present and an entanglement in the time order of the potential events along the closed path.

Without posing restrictions to the thickness 2*T* of the present, we should consider a very fast “sampling rate” of the information (from which the difference between “something happened” or “nothing happened” is evaluated), and then, given a time of traversal Δ*τ* >> 2*T*, we could investigate what is happening while the particle is traversing the circuit.

The description of the space foliation emerging in the thick present through an imaginary time of motion and an information potential related to “open choices” between imaginary points on a CTC may promote an idea of non-locality in the emerging imaginary space through its “thickness in time”.

**Figure 4:**
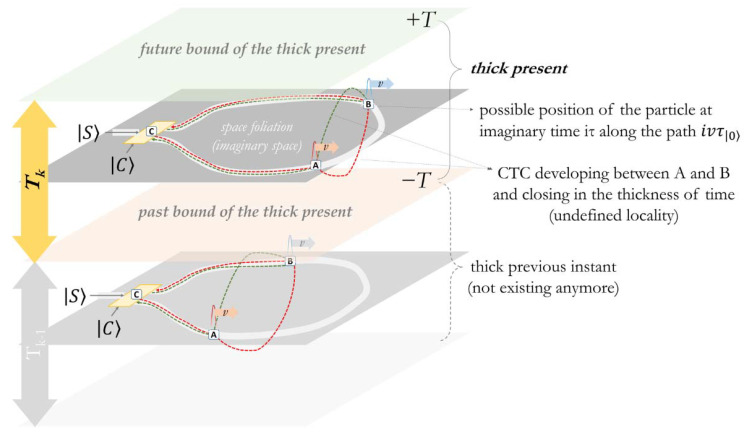
The imaginary space emerges from the information potential in the thick present, and it is described through an imaginary time. CTCs in the thickness are an expression of non-local potential.

The particle, while traversing the circuit, can be considered as propagating in both arms (being in both a forward- and backward-propagating wave), and is potentially on all the points already traversed along the curve in each thick instant. The path is then closed through a CTC developing in the thickness of the present (orthogonal to the space distance axis *ict*), representing a non-local correlation between the entangled possible imaginary locations of the particle within the imaginary space (as in Figure 4).

The relation between the causal non-separability and the cyclicity of the causal structure has also been highlighted in Ref. [43], and a connection between UCO, non-locality, and closed curves in the context of logic games has been investigated in Ref. [44]. Still, a description of the information potential as CTC developing in a time-symmetric thick present seems novel among the interpretations of entanglement. In a QIS perspective, we can consider these CTC@*T_k_* as spacetime “memory-loops”, able to encode the information potential of a logically consistent choice in the current space of events and possibilities, of which the outcome is open at the most fundamental level.

### 4.2. Chasing Non-Local Information

Non-local information shall not allow faster than light communications (intended as a transmission of a message with a non-random information). Moreover, considering a finite speed for the causal propagation of any information and that two experimenters (separated in a space-like way) can make choices of measurement independently of each other, even in the context of entanglement, the Free Will theorem already introduced concludes that the result of any quantum observation cannot be fully determined by anything previous to the experiment.

Given the Free Will theorem and considering the Bell and Kochen–Specker theorems (Refs. [45,46]), we shall remember as well that QM interpretations based on non-locality must be contextual: the value of a variable is determined considering the interaction with the local system involved in the measurement process.

We should consider that it is in the fundamental randomness of the quantum observation that a “faster than light communication” finds its impossibility (even in the case of non-local correlations), and that a logically consistent causality is preserved.

The status that is “instantaneously updated” at the distant location *C* by a measurement on *S* is coherent with the state of *S* but, to a local observer at *C*, appears as determined by a random process, and so unable to carry meaningful information. Still, from a global and logically consistent perspective in ***T_k_***, the identification of a choice represents an information potential encoded and persisting in the superposition of the outcomes.

In the thick present potential ***T_k_***, an entanglement in the time order (as UCO) as well as among particles in different spatial locations (*EPR* pairs) can be equivalent to a CTC, described as a logically consistent “memory-loop” able to encode the information potential of an open choice (offered in the entanglement) that precludes under or over-determined solutions. Loops are, actually, the most basic circuits for information storage, and CTC@*T_k_* can be considered as a spacetime “virtual memory” to encode the potential in ***T_k_***.

The proposed interpretation of an imaginary space in which non-locality is assured thanks to a thickness in time could offer insights into the “*measurement problem*” or the “*collapse of the wave function*”. In the instants in which the outcome of the choice is still open, the information potential propagates as a CTC orthogonal to the imaginary space. It selects one branch of the CTC and defines a causal path in the instant of observation. 

**Figure 5:**
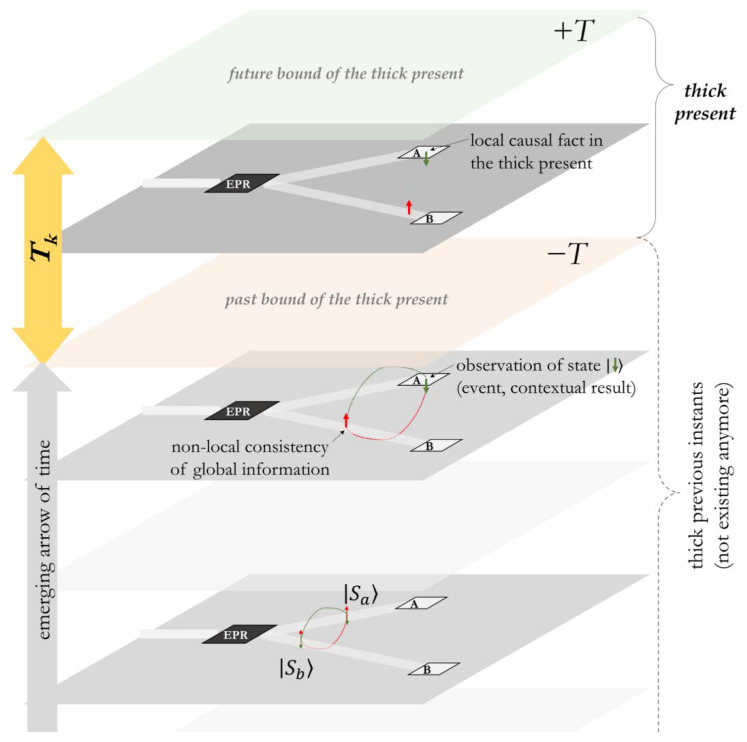
Successive snapshots of the imaginary space foliation, from the EPR pair generation to the spin measurement at Alice’s location. The information potential persists along the successive instants through the entanglement/CTC as long as it is undetermined. When A defines a contextual outcome in her measurement (blue arrow), the state of the particle directed towards B is coherently defined so that the information keeps a global logical consistency within the thick present.

In the case of an *EPR* pair sent to Alice and Bob, the information propagates superposed in the CTC. When Alice choses to observe her particle, she contextually defines a measurement event, which defines a determined orientation in the former CTC that opens in a causal path, ensuring the logical consistency with Bob’s measure (Figure 5).

The given description seems to put on a similar footing a spatial superposition of paths and an entanglement in time expressed as UCO, but this needs further clarification.

Chiribella et al. showed in Ref. [47] that the combination of entanglement-breaking channels in indefinite order can become a perfect quantum channel, while this is not true if the channels are in parallel (spatial superposition), highlighting a possible fundamental difference between space and time.

To understand the results reported in Ref. [47] in the context of a thick present, we should note that a channel from ***S*** to ***R*** is entanglement-breaking “along the path from ***S*** to ***R***”. When the channels are in series but with an undefined order thanks to a controller qubit, we are implicitly assuming that the coherence with the controller has persisted along the time of traversal of both channels (as well as in the connections to go back to ***S*** before entering the next channel), defining coherent imaginary paths, persisting at ***R*** before any measurement on the controller. The resulting CTC in the imaginary time (coherent along the thick present instants even after the transmission over both channels) defines the UCO through the controller qubit and represents the additional quantum resource where the information is encoded identically along the communication (as for the channel $C_{+}$ in [47]). When the channels are in parallel, after the time of traversal there are no CTCs at ***S*** or ***R*** that could be used as an available quantum resource to encode the transmitted information, given that the entanglement on the “*which way*” would only define a causal branch, eventually selecting a single channel which would still be entanglement-breaking.

## 5. Towards a Holographic Perspective

We have proposed a description of spacetime as a space-like foliation emerging from a time-symmetric thick present information potential ***T_k_*** along an imaginary time of motion. This potential represents the information of entanglement, manifested as undefined causality and non-locality and encoded in the thick present as CTC or “memory loops”.

Even if not always in a *Presentism* perspective, several research groups are actively investigating the relations between entanglement, information, and QG.

Holographic theories (introduced by Susskind in Ref. [48]) describe spacetime as emerging from the entanglement among distant quanta of space, encoded in a bulk region of which spacetime is the boundary. Elaborating these concepts in a *Presentism* perspective, we could consider the potential of the present ***T_k_*** as equivalent to a pair of symmetric bulk regions, in a time-symmetric description from (2*k* − 1)*T* to 2*kT* and from (2*k* + 1)*T* to 2*kT*, respectively, of which the space foliation is the common boundary at 2*kT*.

Following the idea of a spacetime emerging from the information of entanglement among the quanta of space, if particles as well can be described as emerging from information, we should consider non-locality in the emerging foliation as the chances of being in multiple points in the imaginary space seemingly simultaneously, as if there were an imaginary *ER bridge* entangling the distant quanta of space. In this sense, quantum tunneling in a space-like foliation should be described as an entanglement in space and a connection through the real fourth dimension of spacetime: the thickness of the current instant, in which everything could be interconnected. Being time-symmetric, this connection and non-local potential is encoded in the present as the superposition of forward and backward waves, from (2*k* − 1)*T* and (2*k* + 1)*T*, respectively, in a resulting CTC which intersects the foliation in the distant entangled quanta of space.

In a thick present potential, the non-local information of a particle expressed in the wave function could be encoded in the superposition of a probabilistic “bundle of CTC” connecting different locations in the imaginary space. This probabilistic ensemble of memory loops encodes the local information potential and embroiders the otherwise flat fabric emerging along the imaginary *ict*. In a spacetime curved by the information of entanglement among the quanta of space, particles could be intended as a “volume of space entangled on a common mode”, encoded in each cycle as a “spacetime local phase” in respect to the reference of action of the present.

The *Presentism* interpretation of spacetime in a holographic perspective introduced in this contribution is proposed as a conjecture. Additional research is needed for a proper mathematical description of ***T_k_*** in terms of the entanglement among the quanta of space, towards a full comprehension of its encoding in the symmetric bulks and of its relation with the gravitational potential.

**Figure 6:**
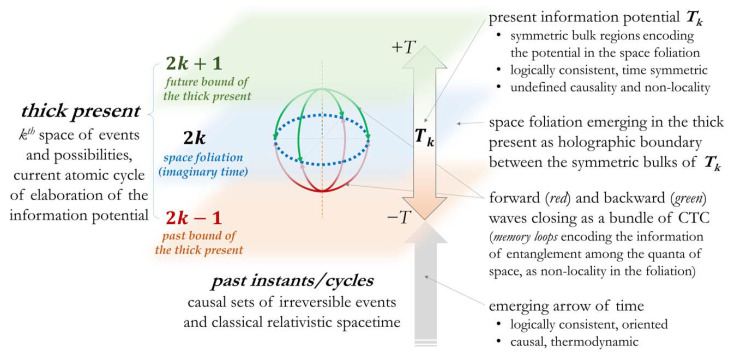
Graphics illustrating the space foliation at 2*kT* in the thick present as the boundary between two symmetric bulk regions extending from (2*k* − 1)*T* to 2*kT* and from (2*k* + 1)*T* to 2*kT*. The bulks encode the information ***T_k_*** (intended in a holographic perspective as an entanglement among quanta of space) from which spacetime emerges. CTCs in the thickness of the present represent the non-local potential; CTC developing in the imaginary time are equivalent to undefined orders of traversed points.

Figure 6 graphically illustrates the holographic description of the thick present proposed, leaving further investigations on this path to a dedicated contribution.

Concluding, extending the famous equation of holographic theories, to represent the possible connection between time (existing as a real thick present potential ***T_k_***), space (emerging in each present instant along an imaginary time), and entanglement (as causally undefined or spatially non-local information of correlation among imaginary quanta of space encoded in the consistent superposition of outcomes of an open choice), we could maybe dare to conjecture, as limited *Flatlanders*, the following synthesis:

$$ER=EPR=CTC@T_{k}$$

## 6. Synthesis and Outlook

In this paper we have discussed an interpretation on the nature of time and a proposal on the relation of entanglement with information, undefined causality, and non-locality.

We have investigated several descriptions of time, connecting elements from the different perspectives in the search for a common intersection, and eventually concluded the existence of a thick present as the only element of reality in an emerging axis of time.

A thick present exists between a causal past of irreversible events and an open future. Within a thick present, we have considered a global information potential ***T_k_*** encoding the *k*th space of events and possibilities through a time-symmetric description, from (2*k* − 1)*T* to (2*k* + 1)*T*. The potential ***T_k_*** has been pictured as a logically consistent information evolving along the present instants, coherent with what happened and what could happen. 

In each instant, spacetime has been described as a space-like foliation emerging from the information in ***T_k_***.

Following a QIS perspective, we have connected the thick present temporal extension to a spacetime “sampling rate” and a discrete elaboration of the information potential, towards the realization of a discrete time, connected to absolute references (such as the Planck units) and not in contrast with a relativistic description of the physics of time as experienced and measured by local observers.

The atomic elaboration of the information in ***T_k_*** has been proposed as an elementary quantum of action and the “fastest event” to evaluate differences, acting as a reference to consistently compare any relativistic perspective on spacetime among observers.

We have concluded the first part of the paper clarifying how a *Presentism* perspective could reconcile philosophy, neuroscience, and physics, and which could be the open challenges towards a quantum description of spacetime based on a thick present potential.

In the second part of this contribution, we have investigated indefinite causal orders, to understand how their information potential could be described and persist along an evolution occurring in thick present instants (given as the only element of reality in time).

Thanks to a parallel with a C-NOT quantum gateway, we have interpreted entanglement as the coherent superposition of the possible outcomes of an “open choice”, in a logically consistent potential that discards under/over-determined solutions.

Following a Paths Integral approach on the circuit implementing the undefined orders, we have described the evolution of the system as superposed imaginary paths developing in opposite directions in the circuit along an imaginary time of motion. Given a time-symmetric description of the potential in the thick present, we have considered these paths equivalent to a forward and a backward wave in the imaginary time of motion, converging at the controller qubit in an imaginary closed path and eventually a CTC.

CTCs are introduced to represent entanglement in both time and space, manifested in the emerging spacetime as the information of undefined causality and non-locality. In a QIS description, CTCs in the thick present have been considered equivalent to “memory-loops” able to encode the information of an open choice of which the outcome is logically consistent and undetermined at the most fundamental level (until observed and contextually defined). When developing along the imaginary time of motion in space, these CTCs represent indefinite orders (of imaginary quanta of traversed space) and an undefined causality. If their path develops through the thickness of the present instant, they encode the non-local correlations in the imaginary space (spatial entanglement).

In the final part of the contribution, we have investigated a possible interpretation of the thick present in a holographic perspective. In the context of a spacetime emerging as flat along an imaginary *ict* and curved by the logically consistent information of entanglement among quanta of space, the description of the potential through CTC in a time-symmetric thick present ***T_k_*** has been conceptually extended to the information expressed in the wave function of an elementary particle.

Massive particles have been interpreted as the potential encoded in a probabilistic bundle of CTCs, that embroiders and deforms the fabric of spacetime, entangling in each present evolution cycle the imaginary quanta of the emerging space foliation.

Following these ideas, we have concluded by conjecturing a possible extension of the famous equation of holographic theories to *ER* = *EPR* = *CTC*@*T_k_*. The additional needed research on this path has been left to a dedicated contribution.

The descriptions of time and of entanglement given in this paper lack explicit mathematical derivations and could be viewed as conjectures inspired by logic and QIS. Nevertheless, the concept of a time-symmetric thick present potential ***T_k_*** that encodes through CTCs the information of entanglement (as undefined causality and non-locality in the emerging space foliation) seems a promising starting framework for the interpretation of our universe in terms of information. The hope is that future works on this path may offer additional insights into the possible ontological nature of information in the emergence of spacetime, towards a proper quantum description of gravity and a more profound understanding of our universe.

## Data Availability

Not Applicable.

## Abbreviations

The following abbreviations are used in the contribution:

C-NOT	*Controlled-NOT* quantum gate. It operates on a quantum register consisting of 2 qubits *C* and *S* (Controller *C* and Target *S*) and flips the qubit *S* if and only if |C⟩=|1⟩. Used to create entangled pairs (*EPR particles*)
CTC	*Closed Time-like Curves*. Closed paths in spacetime. No events occur on their path, and time has no causal or thermodynamic orientation along them. They represent an information potential in the superposition of the outcomes of an open choice (e.g., direction of travel along the curve). When developing between −*T* and +*T* in the thick present, they cross the imaginary time axis in distant quanta of space that result connected (non-locality, entanglement in space). When developing in the imaginary space foliation they represent indefinite orders of traversed quanta of space (undefined causality, entanglement in time). They are considered in the paper as “memory loops”.
EPR	*Einstein–Podolsky–Rosen*. Reference to the 1935 famous contribution on the incompleteness of QM. Intended in the paper as the *EPR paradox*, a synonym of entanglement and entangled particles (*EPR pairs*)
ER	*Einstein–Rosen bridge*. Quantum wormhole connecting far regions of spacetime. Intended in the paper as entanglement between distant quanta in the imaginary space foliation (non-local information potential)
GR	*General Relativity* (theory of)
ICT	*Information and Communication Technologies*
*ict*	*Imaginary coherent time*. Described as the imaginary time of motion (*it*) at the speed of light (*c*) between distant quanta of space. It is proposed in the paper as an imaginary axis of spatial distance and, in the absence of additional information potential, it defines a flat space foliation emerging in the present
OPT	*Operational Probabilistic Theories*. Description of QM from first principles based on Information Theory
PI	*Path Integral* formulation of QM
QG	*Quantum Gravity*
QIS	*Quantum Information Science*
QM	*Quantum Mechanics*
***T_k_***	Quantum potential existing in the thick present (at the current evolution cycle, after 2*kT* instants from the origin of the universe). Time symmetric description from (2*k* – 1)*T* and (2*k* + 1)*T* of the entanglement in space (non-local potential in the imaginary space foliation emerging at 2*kT* along *ict*) and of the entanglement in time (undefined causality and indefinite time orders)
TSVF	*Two State Vector Formalism*. Time-symmetric description of QM
UCO	*Undefined Causal Orders*. Entanglement in time (order)
